# Supplementary material for: Association between shortened maternal and fetal telomere length and abnormal fetal development
Source: PLoS One. 2025 Jul 7;20(7):e0327724. doi: 10.1371/journal.pone.0327724 (PMC12233292; doi:10.1371/journal.pone.0327724)
Supplement: Attachment — Submitted filename: Review _ telomere lenght.doc [file pone.0327724.s001.doc]

The article is interesting, but some adjustments and more detailed elaboration are needed in both the introduction and the discussion of the results.

1- Introduction:

1.1 The phrase "chromosomal or molecular variations" is not sufficiently precise, I recommend using more specific terminology, such as copy number variants (CNVs) for structural alterations and single nucleotide variants (SNVs)

1.2 It would be useful if the authors provided more detailed background on previous studies that investigate the association between telomere length and fetal development.

2- Statistycal analysis

The authors should clarify whether the sample size was sufficient to detect a significant correlation in the regression analysis. Including a justification or a power calculation would strengthen the statistical validity of the results.

3- Results:

3.1- The paper uses the term IUGR, which has been largely replaced in current literature by Fetal Growth Restriction (FGR). I recommend updating the terminology to FGR, in line with recent guidelines in obstetrics and fetal medicine.

3.2- The phrase "All fetuses had normal karyotype and array CGH results (Agilent 180K, threshold 400 kb)" could be interpreted as ambiguous. It is unclear whether the array CGH results were also normal. I recommend clarifying this sentence.

The authors should also point out whether some of these cases underwent NGS analysis (exome sequencing).

3.3: The authors should avoid using the expression in bold: “..prenatal diagnosis for the **108 pregnancies affected with CA**” since only 75 fetuses had congenital anomalies, while the other 33 had severe FGR.

3.4 : In the sentence "Fetuses with MCA had significantly shorter telomeres than those with isolated 13 malformations (P<0.001)," the abbreviation MCA has not been defined earlier in the text. I suggest the authors to use abbreviations only when strictly necessary.

3.5. Please clarify the abbreviation RTL and LT ( or the correct term is TL?) since it was used only once: “We then analyzed maternal age-adjusted TL in each group, which confirmed that maternal **RTL** was significantly shorter…” …These results suggest a potential heritability of **LT**…

4- Discussion

4.1 – The authors should consider discussing the potential implications and limitations of not performing exome sequencing in cases of multisystem and brain anomalies since it is one of the indications of ES in prenatal diagnostic (many references about this topic); and also to discuss better this topic.

4.2 The sentence "Their findings suggest that TL in prenatal samples could be a useful indicator for assessing the risk of developmental anomalies in fetuses" might be somewhat overestimating the role of TL, given that morphological screening in the first trimester already detects around 40% of malformations. I would suggest rephrasing this to reflect that TL could be considered as an additional tool in assessing risk, particularly in maternal samples, rather than a primary indicator.

Figures:

The figures have very blurred lettering. I suggest using a version with better visualization.
